# Bladder/bowel dysfunction in pre-school children following febrile urinary tract infection in infancy

**DOI:** 10.1007/s00467-020-04853-4

**Published:** 2020-12-04

**Authors:** Sofia Sjöström, Ulla Sillén, Marc Bachelard, Ewa Johansson, Per Brandström, Anna-Lena Hellström, Kate Abrahamsson

**Affiliations:** The Pediatric UroNephrology Center, The Queen Silvia Children’s Hospital, The Sahlgrenska Academy at the University of Gothenburg, 416 85 Gothenburg, Sweden

**Keywords:** Bladder/bowel dysfunction, Pyelonephritis during infancy, Urinary tract infection, Vesicoureteral reflux, Follow-up from infancy

## Abstract

**Background:**

An association between bladder-bowel dysfunction (BBD) and urinary tract infection (UTI) is well-known. However, a question less explored is whether children with UTI early in life also have increased prevalence of BBD after they are toilet-trained. In this study, consecutively selected children with pyelonephritis during their first year of life were assessed for BBD at pre-school age.

**Methods:**

Ninety-two children (51 boys) hospitalized due to pyelonephritis during their first year of life were assessed for BBD at median age 5.4 years. A validated BBD questionnaire, along with urine flow and residual volume measurements, was used for diagnosing BBD. During follow-up, the group was well-characterized regarding renal status, vesicoureteral reflux (VUR), and recurrent UTI.

**Results:**

BBD was diagnosed in 35/92 (38%), of which the majority was sub-diagnosed with dysfunctional voiding (DV). There was a strong association between BBD and recurrent UTI during follow-up (*p* < 0.0001), but only a slight association with VUR status at presentation. Nevertheless, in the group with both BBD and VUR, recurrent UTI was four times higher (12/13, 92%) than in children who had neither VUR nor BBD (23%), (*p* = 0.0008). BBD was also associated with kidney damage (*p* = 0.017).

**Conclusion:**

In children with pyelonephritis during the first year of life, 38% had BBD at pre-school age, regardless of whether they had VUR or not. The study shows an important association between BBD and recurrent UTI, so an assessment of BBD is therefore recommended for pre-school children with UTI, especially when they have history of pyelonephritis during infancy.

**Supplementary Information:**

The online version contains supplementary material available at 10.1007/s00467-020-04853-4.

## Introduction

Many studies have shown that bladder-bowel dysfunction (BBD) is a risk factor for UTI, especially in children with dilating VUR. In most of the existing studies, children with bladder dysfunction were investigated for UTI [1, 2], and inclusion in such studies was often restricted to certain high-risk sub-groups [3, 4]. Few studies have prospectively studied a group of young toilet-trained children with UTI and made an association with the presence of BBD. The exception is the RIVUR/CUTIE studies [5, 6]. Both studies included patients with UTI, with and without VUR. Bladder/bowel function was investigated using a validated questionnaire (DVSS) for children who had been toilet-trained [7]. BBD was reported in about half of the patients and was a risk factor for further recurrent UTI, mainly in children with dilating VUR who had not received prophylactic treatment.

Bladder-bowel dysfunction (BBD) is defined as the combination of lower urinary tract dysfunction and constipation [8]. Today, the importance of bowel problems for bladder function is recognized, and the questionnaires used included questions on symptoms from both systems [9, 10]. A consensus view on the evaluation and management of BBD in children with UTI was recently published, highlighting the importance of investigating children with UTI for BBD [11].

The aim of the study was to evaluate the prevalence of BBD in a group of children of pre-school age with a history of pyelonephritis during the first year of life. The group was well-characterized regarding renal status, VUR, and recurrent UTI. The associations between BBD and these three variables, in particular recurrent UTI, were evaluated.

## Patients

A group of 92 children (51 boys) with a median age of 5.4 years (range 4 to 6.8) was included in this prospective follow-up study, with a new evaluation of bladder function and a review of the patient’s medical records. These children had previously participated in a larger study where bladder function was evaluated using videocystometry (VCM) after treatment for pyelonephritis during the first year of life. In this previous study, 151 infants were consecutively included over a 2-year period, after treatment for bacteriologically proven first-time pyelonephritis. The mandatory voiding cystourethrography (VCUG) was carried out together with simultaneous pressure measurement, VCM. Of the infants in the study described, only 92 were available for follow-up of bladder function: 11 had moved out of our area, 34 could not be reached, two had language problems, four had other conditions, and 18 declined participation. No difference between groups, participants in the study versus non-participants, was found regarding kidney damage (*p* = 0.69) or VUR (*p* = 0.54) at presentation.

The criteria for diagnosing acute pyelonephritis during the first year were bacteriological proven UTI, fever ≥ 38.5, and/or poor general condition and with elevated C-reactive protein. Patients with pyelonephritis during the first year of life participated in a structured follow-up program run by the pediatric nephrology department in our Children’s Hospital. Demographics for the 92 children are shown in Table 1. Data for VUR and kidney damage were extracted from medical records. Of the patients, 24 (26%) had VUR at presentation, of whom 16 (19%) had dilating VUR (none with grade 5). In ten of the 24 cases, a second VCUG was performed at mean age of 3.4 years (ranging from 2 to 5 years) and in six of the ten the VUR had resolved at that time. In the remaining cases, there were no VCUG follow-ups. Therefore, only VUR at presentation was used in the analyses. A nephroureterectomy was performed on a patient who had grade 4 VUR and generalized kidney damage with differential kidney function below 10%.

**Table 1:** Demographics in 92 children, with history of pyelonephritis in infancy, and with follow-up of bladder function at mean age 5.4 years

Variable	Boys	Girls	Total
Participants, number	51	41	92
Age at initial febrile UTI during first yearof life, years, median (range) Age at present follow-up	0.3 (0.02–0.9)	0.5 (0.04–0.9)	0.4 (0.02–0.9) 5.4 (4–6.8)
VUR at presentation^1^, number of patients (% of total) - Grades ≤ 2 - Grades 3–4 - Bilateral, number of patients (% of VUR)	12 3 9 4	12 4 8 5	24 (26%) 7 17 9 (37%)
Kidney damage at presentation^2^, number of patients (% of total) - Focal damage - Generalized damage - Bilateral damage (% of kidney damage)	25 15 10 1	14 14 0 2	39 (45%) 29 10 3 (8%)
Kidney damage at follow-up^3^, number of patients (% of total) - Focal damage - Generalized damage - Bilateral damage	15 5 10 0	11 11 0 2	26 (30%) 16 10 2 (8%)
Recurrent UTIs during follow-up			
- Number of patients (febrile and non-febrile) (% of total) - Number of UTIs (febrile and non-febrile) - Number of patients with febrile UTI (% of total) - Number of febrile UTIs	17 (34%) 24 9 (18%) 10	20 (48%) 30 13 (31%) 15	37 (40%) 54 22 (24%) 25
-Number of patients per age-group (febrile and non-febrile)^4^			
• < 1 year • 1–3 years • >3 years	10 11 2	7 12 17	17 (19%) 23 (25%) 19 (21%)

For categorical variables, *n* (%) is presented. For continuous variables, median (min; max) is presented

^1^No patient with VUR grade 5. ^2^Only 86 children investigated with DMSA at presentation. ^3^DMSA follow-up mean 2 years after presentation. ^4^The same patient can be included in more than one age group

Dimercaptosuccinic acid (DMSA) scans were performed in 86 of the children a couple of months after febrile UTI, and in the remaining six cases, parents declined scintigraphy investigations. In this first DMSA, 39 children (45%) were diagnosed with kidney damage. As part of the clinical follow-up, a second DMSA was performed at the age of two, but only in children with kidney damage at presentation, or in children with recurrent febrile UTI. At this time, the total number of children with kidney damage had decreased to 26 (30%), mainly due to the disappearance of discrete focal damage (Table 1). The number of children with generalized kidney damage was unchanged. Kidney damage had not progressed in any of the children with repeat DMSA.

## Methods

### Bladder function studies

In this study, a follow-up of bladder function was performed at pre-school age (median age 5.4 years) in the 92 children described above. Bladder/bowel function was explored using a standardized validated questionnaire, together with uroflowmetry and post-void residual urine assessments. The questionnaire comprised 13 questions in three domains: bladder storage phase, bladder voiding phase, and bowel habits. We use the term bladder/bowel dysfunction (BBD) instead of lower urinary tract dysfunction (LUTD) in this manuscript, as suggested by the International Children’s Continence Society (ICCS) standardization document [8]. A specialized nurse interviewed the parent and child about voiding and bowel habits and completed the questionnaire. Information was also requested regarding the age at which the child was dry both night and day. A physical examination was performed on the same occasion. The questionnaire was validated, and a scoring system developed. All questions (symptoms) are graded from 0 to 3, where 0 represented absence of the symptom and 3 having the symptom every day (Supplement 1). The scale for total scores showed a sum cut-off score of ≥ 7 for BBD. Two sub-scales for investigating the type of dysfunction were identified through factor analysis and were used to describe the overactive bladder (OAB) and dysfunctional voiding (DV). For calculation of OAB and DV, separate formulas with separate cut-offs were used, developed from multivariable models [12].

The uroflowmetry and post-void residual urine study was performed over 4 h and included a minimum of two voiding episodes. An estimation of post-void residual urine volume was made by ultrasound. Functional bladder capacity was estimated using the highest sum of voided volume and post-void residual urine. Bladder capacity was presented in ml and as a percentage of the volume expected for age, according to the formula (30 × (age years + 1) ml). This made comparisons between ages possible. Abnormal values for bladder capacity were ≥ 150% of expected for age, and for residual urine ≥ 20 ml, according to recommendations in the ICCS standardization document [8]. Bell and tower-shaped flow curves were defined as normal, whereas staccato, fractionated, and plateau shapes were defined as abnormal. The flow curves were acceptable for evaluation, provided the voided volume exceeded 50% of expected bladder capacity [8].

To decide whether the child had BBD or not, scores from the questionnaire were used. The sub-diagnosis (OAB and DV) was also made from the questionnaire and thus symptom-based. The flow study was used as a support for BBD if abnormal, but an abnormal flow study alone was not sufficient for a BBD diagnosis, since artifacts may be observed when children void on a uroflowmeter.

### Diagnosis of UTI during follow-up

Medical records from the period between the occurrence of pyelonephritis during infancy and the present follow-up were checked for a history of UTI (febrile or non-febrile), and parents/patients were also asked about such episodes. For a diagnosis of UTI, a bacteriuria level of 100,000 cfu/ml or greater in urine obtained by the midstream or bag technique was required. Febrile UTI (pyelonephritis) was diagnosed when the child had a body temperature ≥ 38.5 °C and with a supporting laboratory test (increased C-reactive protein). Non-febrile infections were registered as cystitis or asymptomatic bacteriuria (ABU), depending on the presence or absence of bladder symptoms. Most of the 17 children with dilating VUR at diagnosis were prescribed antimicrobial prophylaxis for a mean of 2 years. At the present follow-up, none was on prophylaxis.

### Kidney scintigraphy assessments

All DMSA scans were performed according to European guidelines [13]. Kidney damage was classified according to uptake defect (focal, multifocal, or generalized) and split function. In uptake defects, damage was classed as discrete if split function was ≥ 45%, moderate if split function was 40 to 45%, and marked if split function was < 40%. In DMSA follow-up, kidney damage was classified as healed (no damage seen), regression, unchanged, or progression. Thus, a kidney without an uptake defect and split function of 45% or greater was classified as normal, whereas a kidney with decreased or absent uptake in one or more areas was considered abnormal, regardless of split function.

### Statistical methods

Continuous variables are presented showing mean, standard deviation, median, minimum and maximum, and categorical variables with number and percentage. Analyses of recurrent UTIs and VUR/kidney damage were performed using logistic regression with BBD variables and scores as independent variables in univariable models. Event rates, odds ratio with 95% confidence interval, *p* value, and the area under the ROC curve with 95% confidence interval are presented. All tests were two-sided with alpha 5%. The analyses were performed using SAS 9.4, SAS Institute Inc., Cary, NC, USA.

Approval for this study was granted by the institutional review board at the University of Gothenburg, Sweden.

## Results

### Voiding history

**Fig. 1:**
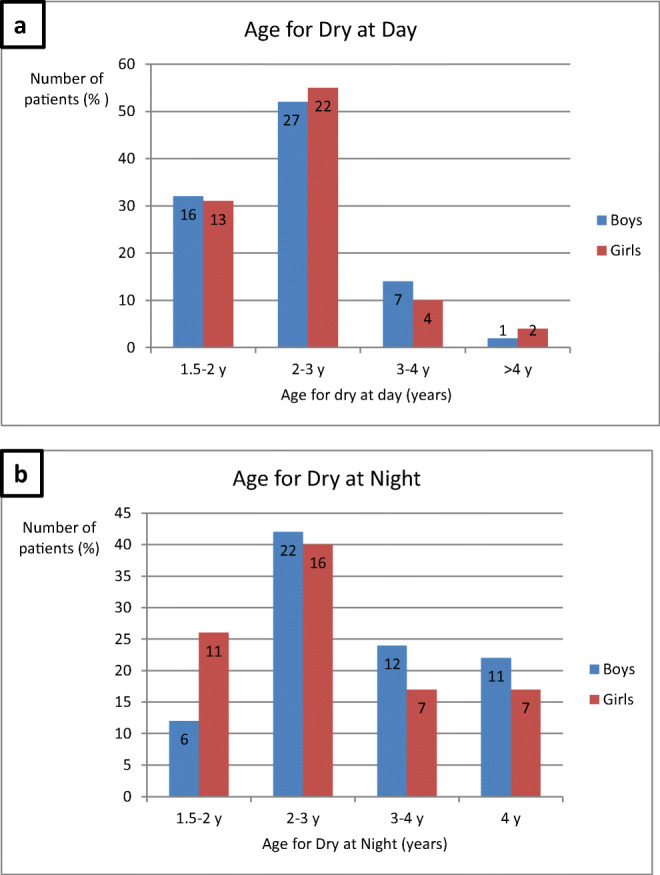
Ages for dry at day (**a**) and dry at night (**b**) in 92 children with a history of pyelonephritis in early infancy. Number of children are given both in % of total number of boys (blue bars) and girls (red bars) and in actual number of children (figure within bars)

The boys and girls became dry during the day at similar ages; one-third of children were younger than 2 years, and about half were between two and three. Only a few children were not dry after the age of four (Fig. 1a). Most children became dry at night between the ages of two and three, with a tendency for boys to become dry later (Fig. 1b). No significant association between the age of dryness and UTI or BBD was found, even if the likelihood of patients with UTI increased in children who were older when becoming dry, which applied to both day (*p* = 0.36) and night (*p* = 0.28).

At follow-up at a median age of 5.4 years, 35 patients (38%) had BBD, according to the bladder-bowel questionnaire (total score ≥ 7). BBD was seen in 19/41 (46%) girls and 16/51 (31%) boys (*p* = 0.21) (Table 2). Overall, a higher BBD score was seen in girls (*p* = 0.054). For numbers of patients with each symptom, see questionnaire in Supplement 1. The age at the latest follow-up did not influence the number of children with BBD. When sub-grouping the dysfunction, 19 patients (54%) had primarily DV, whereas 11 (31%) had OAB as the only sub-diagnosis (Table 2). In another five patients, neither OAB nor DV calculations reached the level for cut-off. These latter patients had low scores for total BBD (scores 7 to 9). Overall, only six of the 35 patients with BBD had constipation problems (17%), and out of the total 92 patients, the number was 13 (14%). Nocturnal enuresis, not included in the questionnaire, was seen in 21 patients (15 boys) of whom 11 also had BBD (8 boys).

**Table 2:** Result of bladder-bowel dysfunction (BBD) assessments in 92 patients with a history of pyelonephritis in early infancy

Variable	
BBD (number of patients)	
No	57 (62%)
Yes (score ≥ 7), - Boys - Girls	35 (38%) 16 (31%) 19 (46%)
BBD yes, score (*n* = 35)	11 (2.2) 11 (7–20)
OAB (number of patients)	
No	24 (69%)
Yes	11 (31%)
DV (number of patients)	
No	16 (46%)
Yes	19 (54%)

For categorical variables *n* (%) is presented

For continuous variables mean (SD)/median (min-max)

Multivariable sub-scales for overactive bladder (OAB) and dysfunctional voiding (DV) for sub-grouping of the dysfunction

### Flow/residual urine measurements (Table 3)

The flow curve was bell shaped in 69 (78%) of the children performing a flow study in the follow-up. The remaining 19 children (22%) had abnormal urinary flow curves, with a staccato (*n* = 10) or fractionated (*n* = 9) shape. No association between abnormal flow curves and BBD was found; although in children with DV, an abnormal flow-curve was seen in 45% (13/29). However, an association was shown between the number of children with an abnormal flow curve and DV (13/19, 68%), when compared with children with a bell-shaped flow curve and DV (16/69, 23%) (*p* = 0.0008). Post-void residual urine was abnormal (≥ 20 ml) in only one patient (1%). Bladder volume at voiding (voided volume + residual urine) was large (≥ 150%) in 15 patients (17%) but was not associated with an abnormal flow curve (*p* = 0.13) or BBD.

**Table 3:** Uroflow/residual urine assessment results vs. BBD (bladder-bowel dysfunction) and the calculated sub-diagnoses OAB (overactive bladder) and DV (dysfunctional voiding) in patients with a history of pyelonephritis in early infancy

Uroflow/residual urine *n *= 88^1^	Total number	BBD (score ≥ 7)		Sub-diagnoses BBD	
		Yes	No	OAB	DV
Shape of flow curve, number of patients (%)					
- Bell	69 (78%)	29 (83%)	40 (75%)	9	16 (55%)
- Staccato (*n* = 10), Fractionated (*n* = 9)	19 (22%)	6 (17%)	13 (25%)	2	13^2^(45%)
Post-void residual urine					
- ≥ 20 ml, number of patients (%)	1 (1%)		1		
Bladder volume at voiding (voided + residual volume)					
- ≥ 150% of expected for age, number of patients (%)	15 (17%)^3^	3 (20%)	12 (80%)		3

^1^Data missing in 4 patients. ^2^Calculated score indicates DV, of these only 3 cases BBD (score ≥ 7) and 10 cases non-BBD (score = 6). ^3^Flow curve abnormal in 6 (40%)

### BBD and VUR (Table 4)

VUR (all grades) was diagnosed during infancy in 24 children (26%) and dilating VUR (grades 3 and 4) in 17 (19%). BBD at follow-up was identified in 13/24 (54%) of children with any grade of VUR, in 9/17 (53%) in dilating VUR, and in 22/68 (32%) in children with no VUR. In assessing the association between VUR and BBD, only a tendency was identified (VUR-grades 1–4, *p* = 0.066, and grades 3–4, *p* = 0.070). The same was found in children with DV and dilating VUR (8/17 children, *p* = 0.070).

**Table 4:**
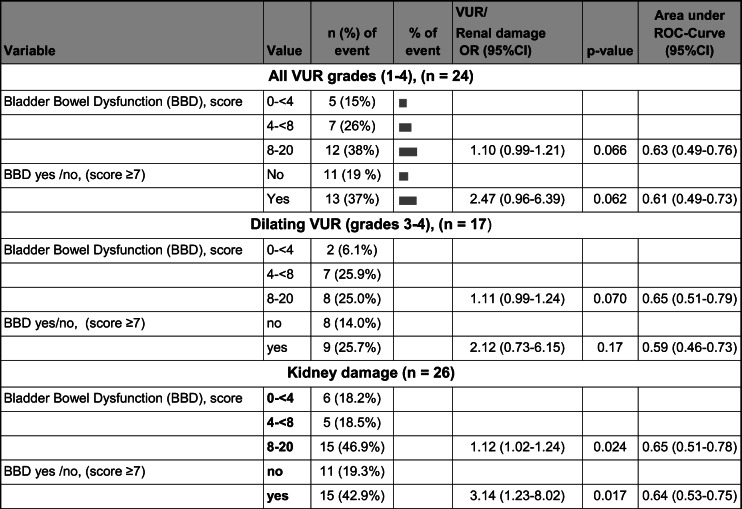
Bladder-bowel dysfunction (BBD) and its association to VUR and kidney damage in 92 children with a history of pyelonephritis in early infancy

### BBD and kidney damage

Kidney damage was seen in 26 children (30%) at the age of two. Of these, 15/26 (58%) were diagnosed with BBD at follow-up, whereas 20/62 (32%) had BBD but no kidney damage. BBD at follow-up is shown in univariate logistic regression analyses to be significantly associated with kidney damage, both in analyses with BBD as a continuous variable (scores, *p* = 0.024) and as a categorical variable (yes/no, *p* < 0.017) (Table 4). However, when performing similar tests using the calculations for OAB and DV, no significant associations were found. At follow-up, when testing only the ten patients with generalized kidney damage, no association was established between BBD and such congenital kidney damage.

### BBD and recurrent UTI (Table 5)

During follow-up (from episode of pyelonephritis in infancy to 5.3-year follow-up), recurrent UTIs were seen in 37 patients (40%) with 54 infections. More than one recurrence was seen in nine patients. Of the 37 children, 22 had febrile UTI with a total of 25 infections (Table 1). The remaining 15 patients had only non-febrile infections (cystitis).

**Table 5:**
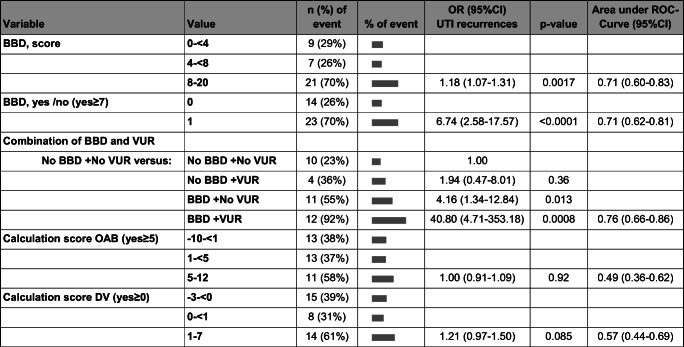
Recurrent urinary tract infection (UTI) and its association to bladder-bowel dysfunction (BBD), to a combination of BBD and VUR, to overactive bladder (OAB) and to dysfunctional voiding (DV)

Of the 37 patients with recurrent UTI, 23 (66%) had BBD at the follow-up study. BBD was associated with recurrent UTI in univariate logistic regression analyses, both with BBD as a continuous variable (scores, *p* = 0.0017) and as a categorical variable (yes/no, *p* < 0.0001) (Table 5). With an increasing number of UTIs per patient, the probability of BBD increased (*p* = 0.0002). When performing the same calculations for the 22 patients with febrile UTI only, similar findings were obtained but the significance of the results was weaker. However, when performing similar tests using the calculations for OAB and DV, no significant associations were found.

### Recurrent UTI versus BBD and VUR together (Table 5)

In children with both BBD at follow-up and VUR diagnosed during infancy, the number of children with recurrent UTI was four times higher (12/13, 92%) than in children with neither BBD nor VUR (10/46, 23%), (*p* = 0.0008). When a child had BBD but no VUR, the risk of UTI increased 2.4 times (11/21, 55%) (*p* = 0.013), but when a child had VUR but no BBD, the risk of developing a UTI did not increase (4/11, 36%), (*p* = 0.36).

## Discussion

In a carefully selected group of children with a history of febrile UTIs during the first year of life, 38% was identified as having BBD at the age of five. To identify BBD, we used a recently validated bladder-bowel questionnaire, and to estimate the type of BBD, we used the sub-scales developed for OAB and DV [12]. We observed that 54% of the children with BBD in our study belonged to the DV group. This was higher than the overall prevalence in another study of patients with bladder dysfunction recruited from a urotherapy group, where the majority had OAB [14]. Our results were more in line with findings regarding bladder dysfunction in VUR patients [3, 15].

DV as a dysfunction with increased pelvic floor activity during voiding should, according to ICCS recommendations, be diagnosed with an abnormal urinary flow [16]. In the present study, DV was diagnosed only with symptoms during the voiding phase, and in the additional flow studies, staccato/fractionated curves were found only in half of the children with DV. We suggest that the often mild voiding symptoms of DV, i.e., not seen on a daily basis, was the explanation for the poor compliance between symptoms and flow, and in addition, the flow study was performed only on one occasion. Other possible explanations are that the DV group in our study included other outflow conditions without the overactivity in the pelvic floor [8, 17]. In addition, there are indications that the association between abnormal flow curves and DV is not as straightforward as has been suggested [17, 18].

Today, the importance of bowel problems for bladder function is recognized, with constipation identified as the main bowel problem causing bladder symptoms [9, 10]. In the present study, constipation was seen only in 17% of the children with BBD, which was similar to urotherapy recruited cohorts [12, 14]. Levels were also close to those found in children evaluated for BBD after UTI (22%), i.e., similar inclusion criteria as in our study [6]. In children with both VUR and BBD, the number of children with bowel problems was higher (50%) [3]. Differences between constipation frequencies were probably explained by the different selection criteria for the groups of children, even if diagnostic criteria for constipation are an important factor. The ICCS recommendation is that the Rome4 criteria [19] should be used for the diagnosis [11].

Previous BBD studies, where UTI was the inclusion criterion and which also used a validated bladder-bowel questionnaire, were the RIVUR/CUTIE studies. Children with UTI and VUR were included in the RIVUR study, randomized to prophylactic treatment or placebo, whereas children with UTI, but without VUR, were included in CUTIE and were not offered any treatment. The RIVUR/CUTIE studies reported a slightly higher frequency of BBD (54%) [6], in children after toilet training age, compared to what was found in our study. An explanation for the difference might be that the RIVUR/CUTIE study included 90% girls, while in our study, boys were in the majority. It has been reported, both in groups of children with known bladder dysfunction [20] and in epidemiological studies [21], that in this age group, more girls than boys have BBD. This tendency was also shown in our study. However, there are epidemiological studies into urinary incontinence in pre-school children showing a similar frequency in boys and girls [22].

One quarter of the patients in our study had VUR at presentation, and more than half of these children had BBD at follow-up (54%), a proportion similar to many other studies [3, 5, 6]. This means that half of the children with BBD in our study did not have VUR. This means that after a febrile UTI, it is important to investigate children without VUR for BBD, in addition to those with VUR. Such a recommendation as put forward by Shaikh et al. [6] is an approach taken by many pediatric urologists [23] and in addition is advocated by the ICCS [11]. Shaikh et al. also suggested that BBD and VUR are not causally related, due to the relatively high number of children with BBD who do not have VUR [6]. We agree that this may apply after toilet training age and to lower grades of VUR. However, we do not believe that this suggestion applies to all VUR children, especially not younger children with high-grade VUR and BBD, where a causal relationship appears very likely [4, 24]. There may be different etiologies for BBD in small children with high-grade VUR and older children, whether they have low-grade VUR, or no VUR.

Our study showed that BBD was associated with a high risk of recurrent UTI, which has also been shown in many previous studies [1, 5, 6, 25]. Children with BBD and any grade of VUR had the highest risk of recurrent UTI in this study (92%), but children with BBD but no VUR still had an increased risk (55%) when compared with children with neither BBD nor VUR (20%), a relationship also seen in the RIVUR/CUTIE studies [5]. It is important to note that neither in their nor in our study did grade of VUR appear to modify the association between BBD and recurrent UTI. Overall, their recurrent UTI figures were slightly lower, which might be explained by the higher number of patients included in their BBD studies. Also, it is probable that our patients belonged to a more severely affected group since all had experienced pyelonephritis during the first year of life, whereas in their study, 50% of patients had experienced the index infection between the ages of one and six [6].

BBD was also associated with kidney damage, which was identified in the present study, but was not seen in any of the groups in the RIVUR/CUTIE studies, as kidney scarring was rare at inclusion in their studies (2 to 3%). Nevertheless, an association between new kidney damage and BBD was reported in their study [26], while in our study, no new damage was established during follow-up.

It is well-known that pyelonephritis during the first 12 months of life is more common in boys than girls [27], and this was the period when our study subjects were identified (hospitalized for febrile UTI). The number of recurrent UTIs in boys and girls during follow-up was also in accordance with what was shown earlier. That is to say, boys dominated during the first 12 months, whereas girls were in the majority after the age of one, and particularly after the age of three when UTI in boys was rare. The higher risk of UTI in girls has been connected to the difference in anatomy, with girls having a short, straight urethra.

The most obvious causal association between BBD and UTI is incomplete bladder emptying with elevated residual urine [2]. The latter can be a consequence of increased activity in the pelvic floor during voiding (DV), or bladder outlet obstruction induced by, for example, rectal distension with compression of the adjacent bladder neck [28]. A connection between overactive contractions inducing pelvic floor activity and UTI has also been shown. Increased uropathogenic organisms in the gastrointestinal tracts may also be responsible for an increase in UTIs, but this is mainly seen when constipation is a part of the BBD [29, 30].

A limitation of the study was the low number of children in the sub-groups identified, especially when it came to testing the variables against each other. Yet significant results were obtained in many aspects. The strength of the study was the homogeneity of the patients and the fact that a validated questionnaire was used for exploring BBD.

## Conclusion

In children of pre-school age who had experienced febrile UTI during the first year of life, more than one third had BBD. Of these BBD cases, one half also had a history of VUR. Recurrent UTI was found in 40% of the cohort but was higher when BBD was present and seen in almost all cases when the patients had both BBD and VUR. Thus it is recommended that, when toilet-trained, young children who had previously experienced febrile UTI are investigated for BBD, especially when they also have recurrent UTIs and/or VUR.

## Supplementary Information

ESM 1 (DOCX 22 kb)

## Data Availability

All the data used in this publication are available through contact with the corresponding author.
