# Supplementary material for: Bladder/bowel dysfunction in pre-school children following febrile urinary tract infection in infancy
Source: Pediatr Nephrol. 2020 Dec 4;36(6):1489–97. doi: 10.1007/s00467-020-04853-4 (PMC8084797; doi:10.1007/s00467-020-04853-4)
Supplement: Supplementary file 1 — (DOCX 22 kb) [file 467_2020_4853_MOESM1_ESM.docx]

| **SCORING** | 3 | | 2 | | 1 | | 0 | |
| --- | --- | --- | --- | --- | --- | --- | --- | --- |
| 1. How often does the child pee during the day? | 1-2 times  1 | | 3 times  4 | | More than 7 times  7 | | 4-7 times  23 | |
| **BEFORE AND AFTER PEEING** |  | |  | |  | |  | |
| 2. How often does the child delay peeing, if it is not reminded? | Every day  20 | | Several times a week  9 | | A few times a month  2 | | Never  4 | |
| 3. Does the child have to rush to the toilet to pee? (sudden, urgent need) | 14 |  | 9 |  | 9 |  | 3 |  |
| 4. Does the child react to this sudden, urgent need to pea by stopping it in some way, like sitting on its heels, pressing with hands, crossing legs, taking small steps and so on? | 10 |  | 6 |  | 7 |  | 12 |  |
| 5. How often does the child pee into its underwear (urinary leakage)? | 15 |  | 0 |  | 12 |  | 8 |  |
| 6. Amount of urinary leakage | Wet to knees/feet  0 | | Through the trousers  2 | | Some drops  25 | | No leakage  8 | |
| **DURING PEEING** |  | |  | |  | |  | |
| 7. Does the child have to wait for peeing to start? (Hesitancy) | Every day  5 | | Several times a week  1 | | A few times a month  10 | | Never  19 | |
| 8. Does the child need to push or press its stomach when peeing? (Straining) | 1 |  | 4 |  | 7 |  | 23 |  |
| 9. Does the child pee in small portions, i.e. it stops and starts (Intermittency) | 5 |  | 4 |  | 8 |  | 18 |  |
| **PASSING STOOLS** |  |  |  |  |  |  |  |  |
| 10. Does the child have bowel (poop) accidents into its underwear? | 0 |  | 1 |  | 2 |  | 32 |  |
| 11. How often does the child pass a stool (poop)? | Less than once a week  0 | | 1 or 2 times a week  4 | | Once every other day  1 | | Once a day or more  30 | |
| 12. Has the child’s stool (poop) been hard during the past month (Bristol 1-2)? | All the time  0 | | More than half the time  3 | | Less than half of the time  1 | | Never  31 | |
| 13. Is the child receiving treatment for constipation with stool softeners or something else? | Yes, for more than one year  0 | | Yes, for less than one year  3 | | Not now, but previously  3 | | Never  29 | |

**Supplement 1**

Bladder/bowel dysfunction in pre-school children following febrile urinary tract infection in infancy.

Pediatric Nephrology

Sjöström S, Sillén U, Bachelard M, Johansson E, Brandström P, Hellström AL and Abrahamsson K

The Pediatric UroNephrology Center, The Queen Silvia Children’s Hospital, The Sahlgrenska Academy at the University of Gothenburg, Gothenburg, Sweden

Corresponding author: ulla.sillen@gu.se

**Bladder-bowel Questionnaire**

The figures in the questionnaire represent each symptom in 35 patients diagnosed with BBD
